# HIV-1 CRF01_AE subtype and HIV-1 DNA level among patients with chronic HIV-1 infection: a correlation study

**DOI:** 10.1186/s12879-020-4785-6

**Published:** 2020-01-21

**Authors:** Tingxia Lyu, Yongsong Yue, Evelyn Hsieh, Yang Han, Ting Zhu, Xiaojing Song, Wei Cao, Wei Lyu, Jianhua Wang, Taisheng Li

**Affiliations:** 1 Department of Infectious Diseases, Peking Union Medical College Hospital, Chinese Academy of Medical Sciences & Peking Union Medical College, Beijing, 100730 China; 2 Section of Rheumatology, Allergy and Immunology, Department of Internal Medicine, Yale School of Medicine, New Haven, CT USA; 3 CAS Key Laboratory of Molecular Virology and Immunology, Institute Pasteur of Shanghai, Chinese Academy of Sciences, Shanghai, China; 4 University of Chinese Academy of Sciences, Beijing, China; 5 Center for AIDS Research, Chinese Academy of Medical Sciences and Peking Union Medical College, Beijing, 100730 China; 6 Clinical Immunology Center, Chinese Academy of Medical Sciences, Beijing, China; 7 School of Medicine, Tsinghua University, Beijing, China

**Keywords:** HIV, CRF01_AE, DNA reservoir, Antiretroviral therapy

## Abstract

**Background:**

The impact of HIV-1 subtype (CRF01_AE and non-CRF01_AE) on HIV-1 DNA levels in HIV-1 chronically infected patients with suppressive antiretroviral therapy (ART) remains poorly understood. To evaluate the correlation of HIV-1 subtype with DNA level, and identify baseline predictors of HIV-1 DNA decay.

**Methods:**

ART-naïve HIV-1-infected patients from two large multi-center studies in China were classified into CRF01_AE and non-CRF01_AE subtype groups. Peripheral blood samples were collected at baseline and week 12, 24, 48 and 96 after ART initiation and total HIV-1 DNA levels were quantified by real-time PCR. HIV-1 DNA levels at week 96 were categorized into high, moderate, and low levels, reflecting HIV-1 DNA ≥ 3, 2–3, ≤ 2 log_10_ copies/10^6^ PBMCs, respectively, and the corresponding proportion of CRF01_AE and non-CRF01_AE subtype were compared. The baseline predictors of low HIV-1 total DNA levels (≤ 2 log_10_ copies/10^6^ PBMCs) at week 96 were evaluated using a logistic regression model.

**Results:**

Compared to the non-CRF01_AE subtypes (*n* = 185), patients with CRF01_AE subtype (*n* = 188) harboured a higher level of HIV-1 DNA (median: 3.19 vs. 2.95 log_10_ copies/10^6^ PBMCs, *P* < 0.001) prior to treatment. After 96 weeks of ART, HIV-1 DNA levels remained higher in the CRF01_AE subtype group (median: 2.63 vs. 2.39 log_10_ copies/10^6^ PBMCs, *P* = 0.002). There was no significant difference in the proportion of patients achieving high (22.3% vs. 14.6%, *P* = 0.054), moderate (59.6% vs. 60.5%, *P* = 0.849) and low levels (18.1% vs 24.9%, *P* = 0.111) between CRF01_AE and non-CRF01_AE groups. In the multivariable analysis, baseline HIV-1 DNA level and CD4^+^ T cell count but not the subtype were independent risk factors for achieving HIV-1 DNA level ≤ 2 log_10_ copies/10^6^ PBMCs.

**Conclusion:**

HIV-1 CRF01_AE subtype is neither correlated with HIV-1 DNA reservoir decline nor a prognostic factor for achieving lower HIV-1 DNA levels (≤ 2 log_10_ copies/10^6^ PBMCs) after ART. However, higher HIV-1 DNA level in HIV-1 CRF01_AE patients should be aroused much attention and strengthen surveillance during ART.

## Background

HIV-1 subtype is associated with disease pathogenesis and progression of AIDS [1]. For example, patients infected with HIV-1 subtype D experience a more rapid progression to AIDS compared with subtypes A and B [2, 3]. Untreated patients infected with HIV-1 CRF01_AE subtype have a shorter median survival than patients of subtype B [4–6]. This finding may be explained by the predominant association of HIV-1 CRF01_AE subtype with CXCR4 rather than CCR5 tropism, or coreceptor switch of R5/X4 with prolonged infection time, ultimately leading to a more rapid disease progression [7, 8].

To date, the HIV-1 DNA reservoir and recovery of replication-competent HIV in antiretroviral therapy (ART) treated patients with undetectable HIV-1 RNA remains the major obstacle in identifying a cure for HIV [9]. The HIV reservoir is influenced by multiple factors, including the clinical stage of HIV infection, CD4^+^ T cell count, CD4/CD8 ratio, degree of immune activation/inflammation, treatment duration, and HIV-associated neurocognitive disorders [10–15]. As a biomarker, HIV-1 DNA level is usually used to evaluate the persistence and dynamics of the HIV reservoir [14]. Moreover, the HIV-1 DNA reservoir in PBMCs prior to initiation of ART can predict viral rebound after treatment interruption, and is independently correlated with disease progression [16, 17].

Although HIV-1 subtype and HIV-1 DNA levels have each been independently associated with disease progression, the correlation between HIV-1 viral subtype and HIV-1 DNA kinetic changes remains unclear. We performed an analysis of data collected as part of two prospective, multi-site cohort studies to explore the influence of CRF01_AE subtype, the predominant subtype in China, on HIV-1 total DNA level across different time points during the 96 weeks after ART, and to identify baseline predictors of achieving low HIV-1 DNA levels (≤ 2 log_10_ copies/10^6^ PBMCs) after 96 weeks of ART.

## Methods

### Subjects

We performed a secondary analysis of stored samples collected from HIV-1 infected patients enrolled in two large multi-centre, open-label clinical trials prospectively evaluating the efficacy and toxicities associated with approved ART regimens available through the China National Free AIDS Treatment Program respectively from 2008 to 2010 (cohort 1, NCT00872417) and 2012–2014 (cohort 4, NCT01844297). The studies were carried out by the China AIDS Clinical Trial Network in 11 different municipalities/provinces in China, including Beijing, Fujian, Guangdong, Guangxi, Henan, Hunan, Liaoning, Shanghai, Shanxi, Sichuan, and Yunnan provinces. The inclusion and exclusion criteria and study-related procedures of these clinical trials have been previously described in detail [18–20].

The additional criteria for patient inclusion in the present study were as follows: completed at least 96 weeks of follow up with HIV-1 RNA levels maintained ≤50 copies/mL after 48 weeks of ART, 12 patients (10 in CRF01_AE group, 2 in non-CRP01_AE group) experienced viral blips (HIV-1 RNA ≤ 200 copies/mL) were allowed in this study.

As part of the parent studies, sociodemographic and clinical data including sex, age, ethnicity, route of transmission, marital status, and smoking history were collected at the time of study enrolment. Baseline CD4^+^ and CD8^+^ T cell counts, HIV-1 RNA load, and HIV-1 DNA level were also measured for all participants. Follow-up evaluations were performed at weeks 0/12/24/48/96. Peripheral blood samples (plasma, whole blood) were collected at each time point and stored at − 80 °C.

### HIV-1 nucleic acid quantification

HIV-1 total DNA was extracted from the peripheral blood by using MagNA Pure LC DNA Isolation Kit and MagNA Pure LC instrument (Roche Molecular Biochemicals, Mannheim, Germany), amplified and quantified using LTR gene primers with a real-time fluorescence-based HIV detection kit (SUPBIO, Guangzhou, China) [21],the reaction system contains: reaction mixture (44.2 μL), enzyme (0.8 μL), HIV-1 DNA (5 μL). Finally, HIV-1 DNA per 10^6^ PBMCs was calculated by dividing the proportion of lymphocytes and monocytes in the routine complete blood count. The quantification range for HIV-1 DNA using this assay was 20 to 5,000,000 copies/10^6^ WBCs.

HIV-1 RNA was extracted from plasma using the QIAamp RNA mini kit (QIAGEN, Hilden, Germany) and then amplified using the COBAS Ampliprep/TaqMan48 real-time RT-PCR Test (Roche Diagnostics, Indianapolis, Indiana, USA). The detection range of HIV-1 RNA was 40 to 10, 000, 000 copies/mL.

### HIV-1 subtype analysis

The HIV Pol gene was amplified with PrimeScript One Step RT-PCR Kit Ver.2 (TaKaRa, Dalian, China) and then sequenced. PCR primers used for sequencing have been reported previously [7]. Subtypes of HIV-1 were then determined through the Recombinant Identification Program (http://www.hiv.lanl.gov/content/sequence/RIP/RIP.html) and confirmed by neighbour joining phylogenetic analysis via sequence alignment of the Pol gene with reference sequences from the Los Alamos National Laboratory (http://www.hiv.lanl.gov/content/index). In order to further confirm the HIV-1 subtype, sequencing of the V3 loop was also carried out in patients (*n* = 76) preliminarily determined by Pol gene sequencing.

### Statistical analysis

The patients’ baseline clinical and demographic characteristics were compared between HIV-1 subtype groups using the Student’s t-test for parametric continuous variables, and Wilcoxon Rank Sum test for non-parametric continuous variables.

Patients were classified into 3 categories according to their HIV-1 DNA levels at 96 weeks (high: ≥ 3 log_10_, moderate: 2–3 log_10_, and low: ≤ 2 log_10_ copies/10^6^ PBMCs). Differences in the proportion of patients in each group by subtype CRF01_AE and non-CRF01_AE were analysed by Chi squared analysis. Univariate and multivariable logistic regression using the forward entry method to assess the correlation between HIV-1 total DNA level at 96 weeks (≤ 2 log_10_ copies/10^6^ PBMCs). Variables for which *P* < 0.2 in the univariate analyses were included in the subsequent multivariable regression analysis.

All analyses were performed using SPSS version 19.0 (IBM Corporation, Armonk, New York, USA) and GraphPad Prism 6.0 (GraphPad Software, Inc. La Jolla, CA, USA). *P* values < 0.05 were considered statistically significant.

## Results

### Population characteristic

Of the total 999 patients enrolled in the two parent studies (499 patients in NCT00872417 and 500 patients in NCT01844297), 373 patients met the inclusion criteria and were classified into CRF01_AE subtype (*n* = 188, 50.4%) and non-CRF01_AE subtype (*n* = 185, 49.6%) groups. The non-CRF01_AE group included subtypes B, C, CRF07_BC, CRF08_BC and URF). The characteristics of the study population are summarized in Table 1. There were no significant differences between the two subtype groups regarding to sex, age, ethnicity, and ART therapy. However, prior to ART initiation, the CRF01_AE group showed significantly higher plasma HIV-1 RNA levels (4.83 vs. 4.49 log_10_ copies/mL, *P* < 0.001), higher total DNA levels (3.19 vs. 2.95 log_10_ copies/10^6^ PBMCs, *P* = 0.001), lower CD4^+^ T cell count (193 vs. 246 cells/μL, *P* = 0.001) and CD4/CD8 ratio (0.23 vs. 0.27, *P* = 0.006) compared with non-CRF01_AE group.

**Table 1:** Sociodemographic and clinical characteristics of the study population, stratified by HIV-1 subtype

	Total *N = 373*	CRF01_AE *n = 188*	Non-CRF01_AE *n = 185*	*P-*value
Sex n(%)				0.429
Male	263 (70.5)	129 (68.6)	134 (72.4)	
Female	110 (29.5)	59 (31.4)	51 (27.6)	
Age years	35 (27–43)	35 (28–43)	36 (27–43)	0.893
Ethnic category n(%)				0.655
Han	321 (86.1)	157 (83.5)	164 (88.6)	
Minority	52 (13.9)	31 (16.5)	21 (11.4)	
Route of Transmission n(%)				0.002
MSM	120 (32.2)	65 (34.6)	55 (29.7)	
Heterosexual	195 (52.3)	106 (56.4)	89 (48.1)	
Others	58 (15.5)	17 (9.0)	41 (22.2)	
Time between diagnosis and ART initiation (years)	0.26 (0.99–1.18)	0.32 (0.11–1.57)	0.22 (0.09–0.97)	0.245
Baseline HIV-1 DNA log_10_ copies/mL	3.0 (2.6–3.4)	3.19 (2.7–3.5)	2.95 (2.6–3.2)	0.000
Baseline plasma viral load log_10_ copies/mL	4.6 (4.2–5.1)	4.83 (4.4–5.2)	4.49 (3.8–4.9)	0.000
Baseline CD4^+^ T cell count cells/μL	214 (129–299)	193 (103–286)	246 (157–206)	0.001
Baseline CD8^+^ T-cell count cells/μL	788 (530–1080)	733 (526–1058)	835 (545–1113)	0.391
Baseline CD4/CD8 ratio	0.24 (0.15–0.37)	0.23 (0.11–0.35)	0.27 (0.18–0.41)	0.006
96-week HIV-1 DNA log_10_ copies/mL	2.53 (2.09–2.91)	2.63 (2.21–2.97)	2.39 (2.00–2.80)	0.002

Continuous variables are expressed as median (interquartile range). Abbreviations: ART, Antiretroviral therapy; PBMCs, Peripheral blood mononuclear cells

### HIV-1 DNA dynamics

**Fig. 1:**
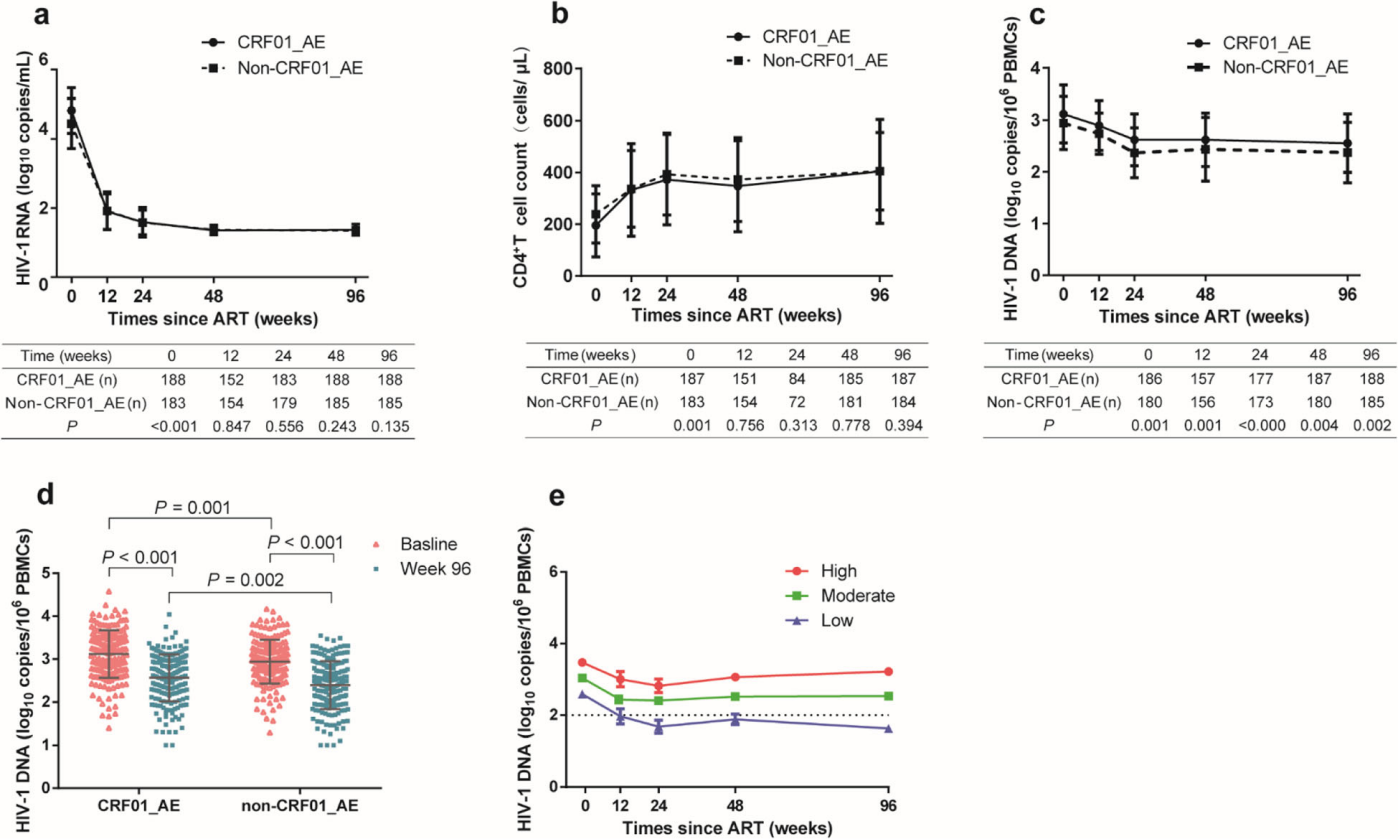
Dynamic changes of clinical parameters during 96 weeks of ART between CRF01_AE and non-CRF01_AE group. **a**. HIV-1 viral load of both groups was controlled efficiently. **b**. Increased CD4^+^ T cell count after ART in both of the two groups. **c**. The HIV-1 DNA level of CRF01_AE is higher than that of non-CRF01_AE group through all the treatment period. **d**. The decay of HIV-1 DNA level at baseline and week 96. **e**. The distribution of individuals classified by HIV-1 DNA level at week 96 (High: HIV-1 DNA level ≥ 3 log10 copies/10^6^ PBMCs; Moderate: HIV-1 DNA level between 2 and 3 log10 copies/10^6^ PBMCs; Low: HIV-1 DNA level ≤ 2 log10 copies/10^6^ PBMCs)

Although HIV-1 RNA levels were equally supressed in both groups, and no significant difference was observed in CD4^+^ T cell recovery over 96 weeks (Figs. 1a & b), the HIV-1 DNA levels remained consistently higher in the CRF01_AE group compared with the non-CRF01_AE group [2.62(2.21–2.97) vs. 2.38(2.00–2.80) log_10_ copies/10^6^ PBMCs at week 96 (*P* = 0.002)] (Figs. 1c & d). The largest decrease occurred during the first 24 weeks of treatment in both groups. At week 24, the median change in HIV-1 DNA from baseline was similar in both groups [− 0.53 (− 0.86 to − 0.28) log_10_ copies/10^6^ PBMCs in the CRF01_AE group vs. − 0.66 (− 1.01 to − 0.29) log_10_ copies/10^6^ PBMCs in the non-CRF01_AE group, *P* = 0.181]. HIV-1 DNA levels plateaued thereafter until week 96 (Fig. 1c).

As pre-ART CD4+ T-cell count and pre-ART HIV-1 DNA showed a strong, negative correlation [22–24], we screened 325 patients (213 from cohort 1, 112 from cohort 4) with a baseline CD4^+^ T ≤ 350 cells/μL to further analysis the relationship between HIV-1 DNA dynamics and the specific antiretroviral regimens. Results showed that the largest decrease in both of cohort 1 and 4 occurred during the first 24 weeks of treatment, the median change from baseline to week 24 was similar in both cohorts [− 0.67 (− 1.03 to − 0.29) log_10_ copies/10^6^ PBMCs in cohort 1 vs. − 0.47 (− 0.73 to − 0.26) log_10_ copies/10^6^ PBMCs in cohort 4, *P* = 0.574] (Additional file 1: Fig. S1a), HIV-1 DNA decline between the two subtypes has also no significant difference in cohort 1 [CRF01_AE group vs. non-CRF01_AE group: − 0.68 (− 1.03 to − 0.29) vs. -0.70 (− 1.01 to − 0.36), *P* = 0.181] (Additional file 1: Fig. S1b) and cohort 4 [− 0.48 (− 0.73 to − 0.25) vs. -0.46 (− 0.78 to − 0.12). *P* = 0.570] (Additional file 1: Fig. S1c).

### The relationship between HIV-1 subtype and HIV-1 DNA level

To analyse the relationship between HIV-1 subtype and HIV-1 DNA level of the overall study population, the DNA levels at week 96 were categorized into high, moderate and low levels with mean total HIV-1 DNA levels of 3.16 (3.12–3.30), 2.53 (2.28–2.73) and 1.73 (1.46–1.91) log_10_ copies/10^6^ PBMCs, respectively (Fig. 1e) and their corresponding distribution were 18.5% (69/373), 60.1% (224/373) and 21.4% (80/373) (Fig. 1e & Table 2). When further stratified by subtype into CRF01_AE and non-CRF01_AE, there was no difference in the proportion of patients in the 3 categories at week 96 (Table 2).

**Table 2:** Category of HIV-1 DNA level at week 96 by subtype

HIV-1 DNA level at 96 weeks n (%)	CRF01_AE *n = 188*	Non-CRF01_AE *n = 185*	*P-value*
High	42 (22.3%)	27 (14.6%)	0.054
Moderate	112 (59.6%)	112 (60.5%)	0.849
Low	34 (18.1%)	46 (24.9%)	0.111

High: HIV-1 DNA ≥ 3 log10 copies/10^6^ PBMCs; Moderate: HIV-1 DNA between 2 and 3 log10 copies/10^6^ PBMCs; Low: HIV-1 DNA ≤ 2 log10 copies/10^6^ PBMCs. Differences in the proportion of patients in each group were analysed by Chi squared analysis

### Association between sociodemographic and clinical risk factors and low HIV-1 DNA level at week 96

In our univariate logistic regression analysis, sex, HIV-1 subtype, baseline CD4^+^ T cell count, CD4/CD8 ratio, and baseline HIV-1 RNA levels were associated with low HIV-1 DNA levels at 96 weeks (Table 3). In the multivariable model, lower baseline HIV-1 DNA level [OR = 0.489 (0.005–0.714), *P* = 0.000] and baseline CD4^+^ T cell count > 200 cells/μL [OR = 3.341 (1.712–6.520), *P* = 0.000] were predictors for achieving a HIV-1 DNA level of ≤2 log_10_ copies/10^6^ PBMCs (Table 3), but not HIV-1 subtype [OR = 1.279, (0.721–2.266), *P* = 0.400] (Table 3).

**Table 3:** Association between clinical risk factors and achieving HIV-1 DNA < 2 log10 copies/10^6^ PBMCs

Variables	Univariate		OR (95% CI)	Multivariate
	OR (95% CI)	*P-value*		*P-value*
Sex				
Male	1		1	
Female	1.758 (1.045–2.957)	0.034	1.447 (0.725–2.885)	0.294
Age years				
18–35	1		1	
36–50	1.439 (0.594–3.487)	0.421	1.328 (0.499–3.534)	0.570
> 50	0.850 (0.335–2.159)	0.733	0.801 (0.291–2.206)	0.668
Transmission route				
Homosexual	1		1	
Heterosexual	2.789 (1.009–7.715)	0.048	3.024 (0.983–9.302)	0.54
Others	3.558 (1.345–9.407)	0.011	2.961 (1.017–8.622)	0.05
HIV-1 subtype				
CRF01_AE	1		1	
Non-CRF01_AE	1.554 (0.941–2.569)	0.085	1.279 (0.721–2.266)	0.400
Baseline CD4 T cell count cells/μL				
< 200	1		1	
> 200	4.903 (2.634–9.124)	0.000	3.341 (1.712–6.520)	0.000
Baseline CD4/CD8 ratio				
< 0.5	1		1	
> 0.5	1.948 (0.953–3.979)	0.067	1.475 (0.663–3.282)	0.341
Baseline HIV-1 DNA log 10 copies/10^6^ PBMCs	0.380 (0.261–0.555)	0.000	0.489 (0.335–0.714)	0.000
Baseline HIV-1 RNA log 10 copies/10^6^ PBMCs	0.758 (0.590–0.974)	0.000	0.985 (0.722–1.344)	0.923

Abbreviations: OR, Odds ratio; CI, Confidence interval; ART, Antiretroviral therapy; PBMCs, Peripheral blood mononuclear cells

## Discussion

This study explored the effect of HIV-1 CRF01_AE subtype on HIV-1 DNA levels over a 96-week period. Our results demonstrate that HIV-1 CRF01_AE infected patients harbour a higher level of HIV-1 DNA throughout the 96-week period compared with patients with other subtypes. However, CRF01_AE subtype does not appear to affect HIV-1 DNA reservoir decline. After 96 weeks, approximately 20% of patients in both groups achieved HIV-1 DNA level ≤ 2 log_10_ copies/10^6^ PBMCs, and HIV-1 subtype was not found to be an independent risk factor for predicting low HIV-1 DNA levels at week 96. Previous studies mainly focused on the impact of viral subtype on the level of HIV-1 RNA and CD4^+^ T cell count, but rarely on HIV-1 DNA [7, 8]. To our knowledge, this is the first national multicentre prospective cohort study in the world to assess the relationship of subtype and HIV-1 DNA reservoir after ART [25].

The median HIV-1 DNA level of our patients at baseline was ∼3 log_10_ copies/10^6^ PBMCs. We observed a period of more rapid decline in HIV-1 DNA during the first 24 weeks of ART, followed by a plateau through week 96, a finding which is consistent with previous studies [22, 26]. This pattern of change is likely due to more rapid degradation of linear HIV-1 DNA present in the early period of ART initiation compared with slower and more difficult elimination of integrated HIV-1 DNA that forms the HIV-1 reservoir later in the course of treatment [27, 28]. HIV-1 DNA level is higher among patients with the CRF01_AE subtype before and after ART (Fig. 1c) and a small gap remains at week 96, therefore, higher pre-treatment HIV-1 DNA level in HIV-1 CRF01_AE patients should be aroused much attention and strengthen surveillance during ART.

Lower HIV-1 DNA levels have been shown to be predictive of a longer period to viral rebound after ART interruption [29, 30]. Despite the reduction in HIV-1 DNA levels observed in our study, approximately 20% patients in both groups were able to achieve a low level (median 1.73 log_10_ copies/10^6^ PBMCs), this reservoir level is similar to post-treatment controllers in the VISCONTI cohort (French), 14 post-treatment HIV-1 controllers with a long-term virological remission after early treatment interruption among 70 patients, and this 14 patients harbour a lower DNA reservoir (median 1.71 log_10_ copies/10^6^ PBMCs) [29]. Because lower HIV-1 DNA level associated with a longer time to viral rebound, therefore, we predicted that 21.4% patients (low DNA category patients in two groups) in our study maybe preferred candidates for further study such as structured treatment interruption.

Total HIV-1 DNA level is also a major predictor of progression to AIDS. A study followed 383 patients in the Seroconverter Cohort (SEROCO, a French prospective cohort) study for more than 8 years, and found the relative risk of progression to AIDS for each 1-log_10_ increase in HIV-1 DNA level was 3.2 [31], while higher CD4^+^ T cell level [32], higher CD4/CD8 ratio, and a lower level of HIV RNA before treatment predicted a more robust therapeutic effect [14]. In our study, higher baseline CD4^+^ T cell count and lower baseline HIV-1 DNA were associated with achieving a low HIV-1 DNA level after ART, which is consistent with previous studies [22, 33]. Furthermore, the time of HIV-1 infection to treatment initiation has been reported as a factor influencing decay total HIV DNA levels during ART [34, 35]. In our study, although the HIV-1 DNA level in both groups declined significantly after ART (Figs. 1c&d), the CRF01_AE subtype exhibited a higher level at baseline, which may explain the higher DNA levels observed at week 96 (Fig. 1d). In contrast, Lu performed a study of 48 patients in Guangxi province (China), followed for 18 months and found the decline in total HIV-1 DNA was significantly faster among patients with the CRF01_AE subtype than in those with subtypes B and CRF07_BC [25]. The ratio of CXCR4-usage for clones from CRF01_AE in our study was higher than that of non-CRF01_AE subtypes (Additional file 1: Table S1), which is similar to Lu’s study. As to the reason why HIV-1 DNA level of 96 weeks of ART in CRF01_AE subtype is in contrast with those of Lu et al. [25], other parameters such as the difference of baseline characteristic or HLA typing might also contribute to the discrepancy.

CRF01_AE has been shown to be associated with a lower CD4^+^ T cell count and higher rates of CXCR4 co-receptor [7, 8, 36]. In our study, baseline CD4^+^ T cell count was lower in the CRF01_AE group than the non-CRF01_AE group. Similar to a previous study [37], the CD4^+^ T cell count in both groups increased sharply after ART, reaching a similar level at week 12 (Fig. 1b). At the same time, baseline CD4^+^ T cell count and HIV-1 DNA level but not the viral subtypes, were predictors for achieving HIV-1 DNA ≤ 2 log_10_ copies/10^6^ PBMCs, which is in agreement with previous studies [33]. The influence of subtype specific differences on disease progression may play a less prominent role in HIV-DNA levels after ART initiation [38].

### Limitations

This study has a few limitations that warrant mention. First, patients were classified into CRF01_AE and non-CRF01_AE groups, without evaluating the impact of other subtypes. We haven’t conduct V3 sequence in whole patients and without follow-up sequences. In addition, other factors, which likely contribute to disease progression, such as immune activation, human leukocyte antigen genotypes, as well as HIV-1 specific cytotoxic T lymphocyte response were not analysed in this study.

## Conclusions

Patients infected with HIV-1 CRF01_AE subtype had higher HIV-1 DNA levels at baseline, but a similar pattern of decline in HIV-1 DNA levels during 96 weeks of ART. Baseline CD4^+^ T cell count and HIV-1 DNA level were associated with achieving HIV-1 DNA level ≤ 2 log_10_ copies/10^6^ PBMCs at week 96, but not HIV-1 subtype. Further research is warranted to explore the relationship between HIV-1 DNA levels in other HIV-1 subtypes and reservoir persistence.

## Supplementary information

**Additional file 1: Figure S1.** HIV-1 DNA dynamics stratified by ART therapy and subtype. **Table S1.** Predicted co-receptor usage based on V3 loop sequence (*n* = 76)

## Data Availability

The datasets used in the current study are available from the corresponding author on reasonable request.

## Abbreviations

ART: Antiretroviral therapy
CI: Confidence interval
HIV: Human immunodeficiency virus
IQR: Interquartile range
PBMC: Peripheral blood mononuclear cells
