# Supplementary material for: HIV-1 CRF01_AE subtype and HIV-1 DNA level among patients with chronic HIV-1 infection: a correlation study
Source: BMC Infect Dis. 2020 Jan 21;20:66. doi: 10.1186/s12879-020-4785-6 (PMC6975045; doi:10.1186/s12879-020-4785-6)
Supplement: Supplementary file 1 — Additional file 1: Figure S1. HIV-1 DNA dynamics stratified by ART therapy and subtype. Table S1. Predicted co-receptor usage based on V3 loop sequence (n = 76) [file 12879_2020_4785_MOESM1_ESM.docx]

**Supplemental Information**

**HIV-1 CRF01_AE subtype and HIV-1 DNA level among patients with chronic HIV-1 infection: a correlation study**

*Tingxia Lyu^1^, Yongsong Yue^1^, Hsieh Evelyn^1,2^, Yang Han^1^, Ting Zhu^1^, Xiaojing Song^1^, Wei Cao^1^, Wei Lyu^1^, Jianhua Wang^3,4^, Taisheng Li^1,5,6,7^*

**Supplemental tables**

Table S1. Predicted co-receptor usage based on V3 loop sequence (n=76).

**Supplemental figures**

Fig. S1. HIV-1 DNA dynamics stratified by ART therapy and subtype.

**Table S1.** **Predicted co-receptor usage based on V3 loop sequence.**

| Subtype | R5 | R5/X4 | X4 |
| --- | --- | --- | --- |
| CRF01_AE (n=32) | 12 (37.5%) | 12 (37.5%) | 8 (25%) |
| non-CRF01_AE (n=44) | 39 (88.6%) | 3 (6.8%) | 2 (4.5%) |

**Fig. S1.**

**
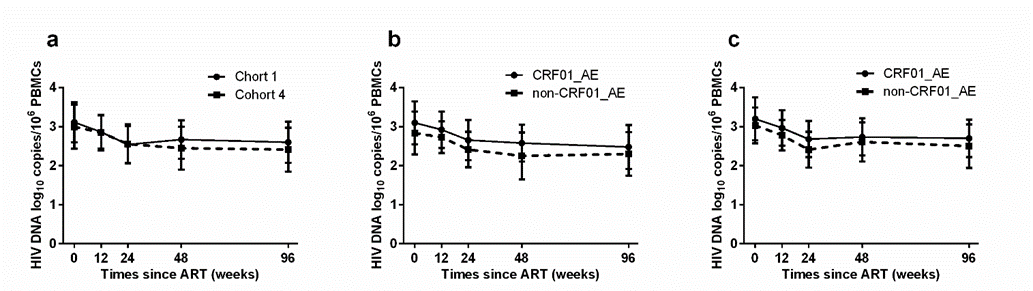
**

Fig. S1. HIV-1 DNA dynamics stratified by ART therapy and subtype. (a). The HIV-1 DNA dynamics in patients with baseline CD4 ≤ 350 cells/μL of cohort 1 and cohort 4. (b & c). The HIV-1 DNA dynamics of CRF01_AE and non-CRF01_AE group in cohort 1(b) and cohort 4 (c).
